# The Post-Storage Performance of RBCs from Beta-Thalassemia Trait Donors Is Related to Their Storability Profile

**DOI:** 10.3390/ijms222212281

**Published:** 2021-11-13

**Authors:** Alkmini T. Anastasiadi, Efthymios C. Paronis, Vasiliki-Zoi Arvaniti, Athanasios D. Velentzas, Anastasia C. Apostolidou, Evangelos G. Balafas, Monika Dzieciatkowska, Nikolaos G. Kostomitsopoulos, Konstantinos Stamoulis, Issidora S. Papassideri, Angelo D’Alessandro, Anastasios G. Kriebardis, Marianna H. Antonelou, Vassilis L. Tzounakas

**Affiliations:** 1 Department of Biology, School of Science, National and Kapodistrian University of Athens (NKUA), 15784 Athens, Greece; alkanast@biol.uoa.gr (A.T.A.); vazoarvaniti@gmail.com (V.-Z.A.); tveletz@biol.uoa.gr (A.D.V.); ipapasid@biol.uoa.gr (I.S.P.); 2 Center of Clinical, Experimental Surgery & Translational Research, Biomedical Research Foundation, Academy of Athens (BRFAA), 11527 Athens, Greece; eparonis@bioacademy.gr (E.C.P.); apostolidou@bioacademy.gr (A.C.A.); vbalafas@bioacademy.gr (E.G.B.); nkostom@bioacademy.gr (N.G.K.); 3 Department of Biochemistry and Molecular Genetics, School of Medicine, Anschutz Medical Campus, University of Colorado, Aurora, CO 80045, USA; monika.dzieciatkowska@ucdenver.edu (M.D.); angelo.dalessandro@ucdenver.edu (A.D.); 4 Hellenic National Blood Transfusion Centre, Acharnes, 13677 Athens, Greece; kostas.stamoulis@gmail.com; 5 Laboratory of Reliability and Quality Control in Laboratory Hematology (HemQcR), Department of Biomedical Sciences, School of Health & Welfare Sciences, University of West Attica (UniWA), 12243 Egaleo, Greece; akrieb@uniwa.gr

**Keywords:** red blood cells, blood transfusion, beta-thalassemia, biomarkers, donor variation, post-transfusion recovery, mouse model

## Abstract

Blood donors with beta-thalassemia traits (βThal^+^) have proven to be good “storers”, since their stored RBCs are resistant to lysis and resilient against oxidative/proteotoxic stress. To examine the performance of these RBCs post-storage, stored βThal^+^ and control RBCs were reconstituted in plasma donated from transfusion-dependent beta-thalassemic patients and healthy controls, and incubated for 24 h at body temperature. Several physiological parameters, including hemolysis, were evaluated. Moreover, labeled fresh/stored RBCs from the two groups were transfused in mice to assess 24 h recovery. All hemolysis metrics were better in the group of heterozygotes and distinguished them against controls in the plasma environment. The reconstituted βThal^+^ samples also presented higher proteasome activity and fewer procoagulant extracellular vesicles. Transfusion to mice demonstrated that βThal^+^ RBCs present a marginal trend for higher recovery, regardless of the recipient’s immune background and the RBC storage age. According to correlation analysis, several of these advantageous post-storage characteristics are related to storage phenotypes, like the cytoskeleton composition, low cellular fragility, and enhanced membrane proteostasis that characterize stored βThal^+^ RBCs. Overall, it seems that the intrinsic physiology of βThal^+^ RBCs benefits them in conditions mimicking a recipient environment, and in the circulation of animal models; findings that warrant validation in clinical trials.

## 1. Introduction

Heterozygotes for beta-thalassemia mutations (βThal^+^) constitute a significant percentage of the general population in several Mediterranean, Sub-Saharan African, and Southeast Asian areas, and also represent a non-negligible proportion of eligible blood donors [1,2,3]. The unique physiology of their red blood cells (RBCs) regarding geometry and cation permeability [4], and the slightly elevated oxidative stress these cells encounter due to imbalanced globin chain synthesis [5], have unexpectedly proven to be advantageous for withstanding the storage lesion under blood bank conditions. Stored RBCs from βThal^+^ donors are exceptional in terms of spontaneous hemolysis, osmotic, and mechanical fragility [1], probably owing to the differential expression of several membrane and cytoskeleton proteins [6] that may provide resistance to lysis [7]. Moreover, the levels of diverse metabolic (e.g., allantoin) [1], protein (membrane-bound antioxidant enzymes) [6], and physiological (membrane protein carbonylation and end-of-storage reactive oxygen species (ROS) accumulation) [1] variables suggest the existence of a superior antioxidant defense system in stored βThal^+^ RBCs. The abovementioned redox parameters seem to be strongly connected to an equally remarkable proteostasis network working mainly at the membrane in situ [8].

In the last years, many studies have shown that donors’ genetic and non-genetic features may not only impact the storability of the donated RBCs but also their post-transfusion efficacy [9]. For instance, blood units from females appear to be superior in terms of hemolysis [10] and redox [11] variables during storage but can be life-threatening in sex miss-matched transfusions [12]. Another example are glucose-6-phosphate dehydrogenase (G6PD) deficient individuals, who while being adequate “storers” [13,14,15], are quite poor “donors” according to both in vitro and in vivo studies [13,16]. In the same context, the RBCs from obese donors are prone to storage lesions and exhibit a trend towards lower recovery in mice [17]. The ever-growing list of donor-related effects on storage capacity and recovery highlighted the need to identify potential biomarkers of transfusion efficacy in fresh and stored blood [18,19,20,21]. Since RBCs from βThal^+^ present a superior storability profile, the aim of this study was to unravel (a) their physiological features when exposed to a potential recipient’s environment in vitro and their recovery when transfused to animal models; as well as (b) potential links between their storage and post-storage states.

## 2. Results

### 2.1. Exposure of Stored RBCs to Plasma at Body Temperature

**Figure 1:**
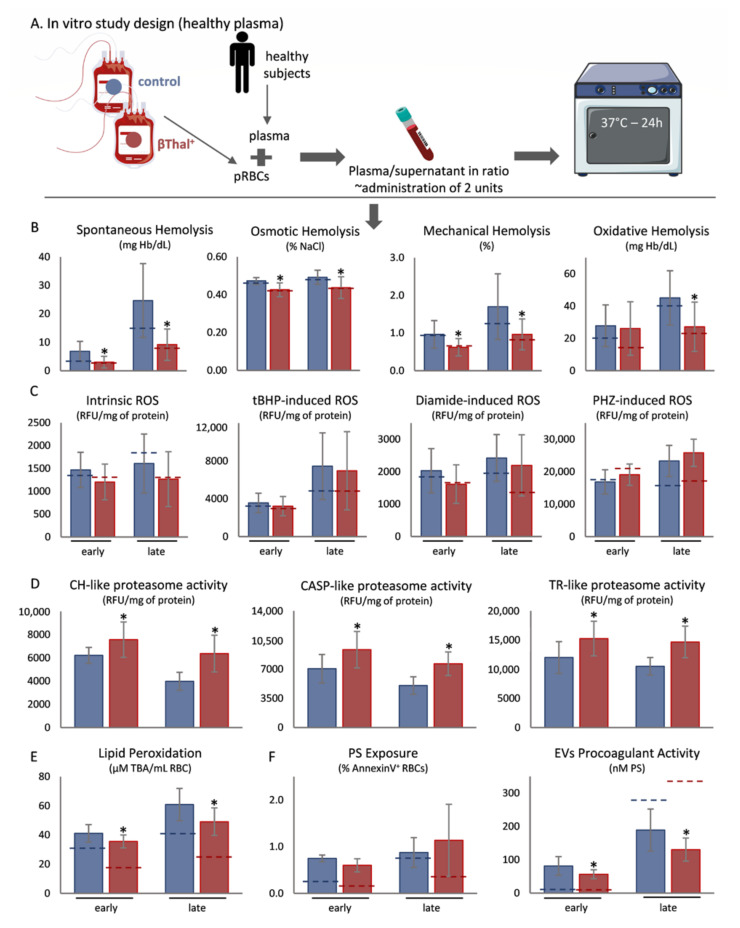
Physiological aspects of beta-thalassemia trait (βThal^+^) RBCs upon exposure to healthy plasma at body temperature. (**A**) Experimental design. (**B**) Hemolysis parameters, (**C**) intracellular reactive oxygen species (ROS), (**D**) proteasome activity, (**E**) membrane lipid peroxidation and (**F**) phosphatidylserine (PS) exposure on RBCs and extracellular vesicles (EVs), between control and βThal^+^ reconstituted RBCs (*n* = 10 per group). (*) *p* < 0.05, βThal^+^ vs. control reconstituted RBCs. Dashed lines: average levels of stored RBCs at 4 °C. tBHP: tert-butyl hydroperoxide; PHZ: phenylhydrazine; CH: chymotrypsin; CASP: caspase; TR: trypsin; TBA: thiobarbituric acid.

**Figure 2:**
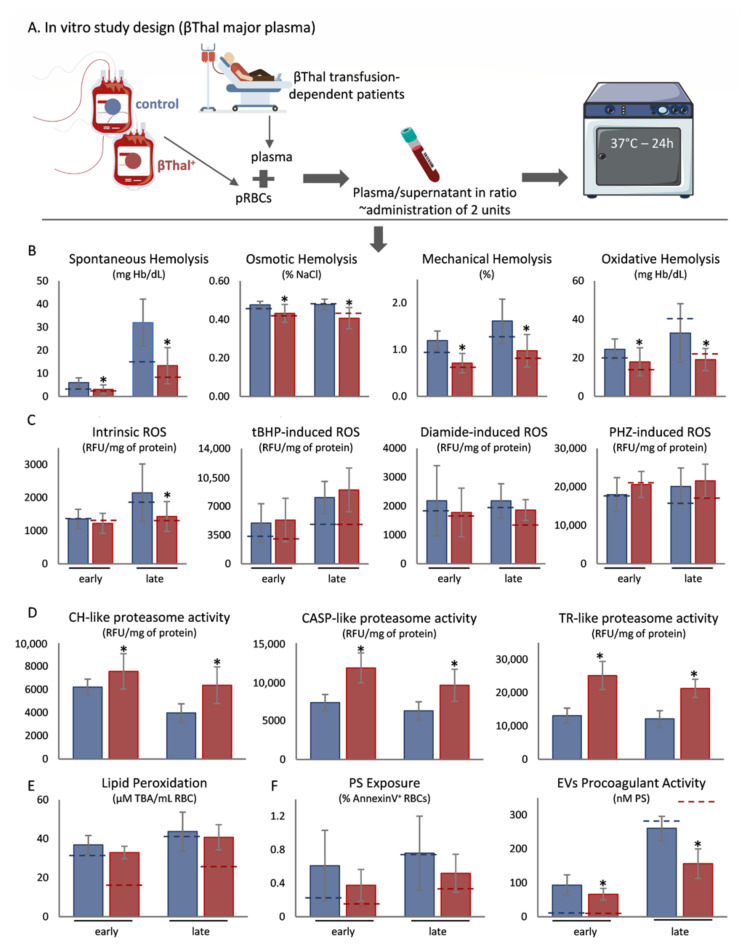
Physiological aspects of beta-thalassemia trait (βThal^+^) RBCs upon exposure to beta-thalassemic plasma at body temperature. (**A**) Experimental design. (**B**) Hemolysis parameters, (**C**) intracellular reactive oxygen species (ROS), (**D**) proteasome activity, (**E**) membrane lipid peroxidation and (**F**) phosphatidylserine (PS) exposure on RBCs and extracellular vesicles (EVs), between control and βThal^+^ reconstituted RBCs (*n* = 10 per group). (*) *p* < 0.05, βThal^+^ vs. control reconstituted RBCs. Dashed lines: average levels of stored RBCs at 4 °C. tBHP: tert-butyl hydroperoxide; PHZ: phenylhydrazine; CH: chymotrypsin; CASP: caspase; TR: trypsin; TBA: thiobarbituric acid.

To study the effects of plasma and body temperature upon stored βThal^+^ RBCs, we used an in vitro model (Figure 1A and Figure 2A) that mimics part of the recipient’s environment. In comparison to controls, βThal^+^ stored RBCs exhibited lower levels of spontaneous, mechanical, osmotic, and oxidative hemolysis when exposed to either healthy (Figure 1B) or beta-thalassemic (Figure 2B) plasma, following both early and late storage. According to receiver operating characteristic (ROC) curve analysis, the osmotic, mechanical, storage and oxidative (in late-stored RBCs) types of hemolysis have very good potential to predict the βThal^+^ status regardless of the plasma environment (Figure S1). Concerning redox-related parameters, while intracellular ROS were equal between the two groups in healthy plasma (Figure 1C), membrane lipid peroxidation was minor in the group of βThal^+^ (Figure 1E). The pattern was slightly different in the disease environment, where intrinsic intracellular ROS presented lower values in late stored βThal^+^ RBCs, along with a trend (*p* > 0.05) in diamide-induced ROS (Figure 2C), but lipid peroxidation did not differ from controls (Figure 2E). All three proteasome activities were greater in the same group, regardless of plasma background (Figure 1D and Figure 2D). Lastly, while phosphatidylserine (PS) exposure was equal between the two groups, PS^+^ extracellular vesicles (EVs) were fewer in the heterozygous reconstituted RBCs in both plasma conditions (Figure 1F and Figure 2F).

### 2.2. Correlations between Storage and Post-Storage Metrics

**Figure 3:**
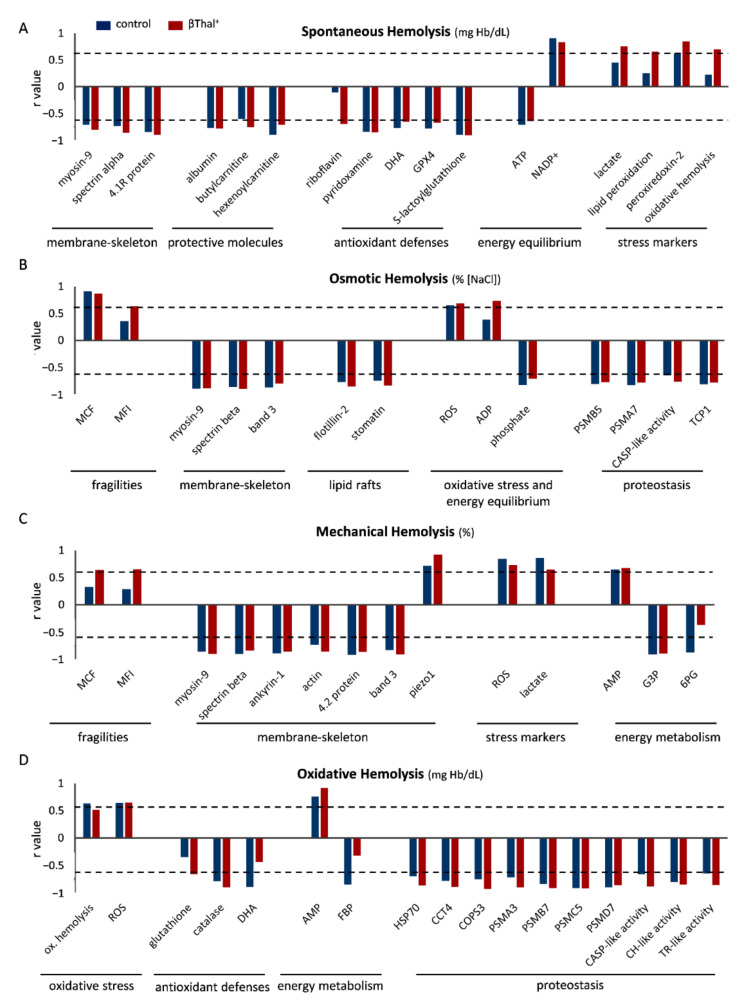
Correlations of storage variables (horizontal axis) with hemolysis parameters post-mixing with plasma. Parameters correlating with (**A**) spontaneous, (**B**) osmotic, (**C**) mechanical, and (**D**) oxidative hemolysis of late-stored control and beta-thalassemia trait (βThal^+^) reconstituted RBCs in thalassemic plasma (*n* = 10 per group) are shown. Similar correlations with slightly different r values emerged in all conditions tested. Dashed line: statistical threshold (*p* < 0.05). 6PG: 6-phosphogluconate, CASP: caspase, CCT: T-complex subunit, CH: chymotrypsin, COPS: COP9 signalosome subunit, DHA: dehydroascorbate, FBP: D-fructose 1 6-bisphosphate, G3P: glyceraldehyde 3-phosphate, GPX4: glutathione peroxidase 4, MCF: mean corpuscular fragility, MFI: mechanical fragility index, ox. hemolysis: oxidative, PSM: proteasome subunit, TCP1: T-complex protein 1, TR: trypsin.

We then sought to search for links between the storage and post-reconstitution profiles of βThal^+^ and control RBCs. Interestingly, components of the cytoskeleton, such as spectrin, as well as a variety of carnitines and albumin negatively correlated with post-reconstitution hemolysis in both groups (Figure 3A). This was also the case for antioxidant molecules and intracellular ATP, while the opposite pattern was observed for NADP^+^ and several stress markers, like lactate (in βThal^+^) and membrane-bound peroxiredoxin-2 (Figure 3A). Osmotic hemolysis of reconstituted RBCs was proportional to that of storage in both donor-groups, and further related to mechanical hemolysis in heterozygotes (Figure 3B). Once again, correlation with membrane and cytoskeletal parameters, along with raft components, was universally evident. While the storage levels of intracellular ROS and ADP (in βThal^+^) presented a positive correlation with osmotic hemolysis, the presence of proteostatic molecules in the membrane resulted in negative correlations (Figure 3B). Moving on, both osmotic and mechanical fragilities of stored βThal^+^ RBCs positively correlated with mechanical hemolysis post-mixing, while many members of the cytoskeleton, such as myosin-9, and the mechanosensitive channel piezo-1 presented negative or positive correlations, respectively, in both groups (Figure 3C). Stress markers (e.g., lactate) and energy depletion (e.g., AMP) had further positive correlations with mechanical fragility, while the presence of metabolites of glycolysis, like glyceraldehyde 3-phosphate demonstrated the opposite link (Figure 3C). Finally, even though storage ROS levels were positively associated with oxidative hemolysis post-reconstitution, regardless of the donor, the oxidative hemolysis storage and post-mixing levels were intra-correlated only in the group of controls (Figure 3D). Antioxidant molecules, like catalase, and an abundance of proteostasis-related proteins (e.g., proteasome subunits, HSPs) were inversely associated with oxidative hemolysis, while AMP levels offered a positive association (Figure 3D).

**Figure 4:**
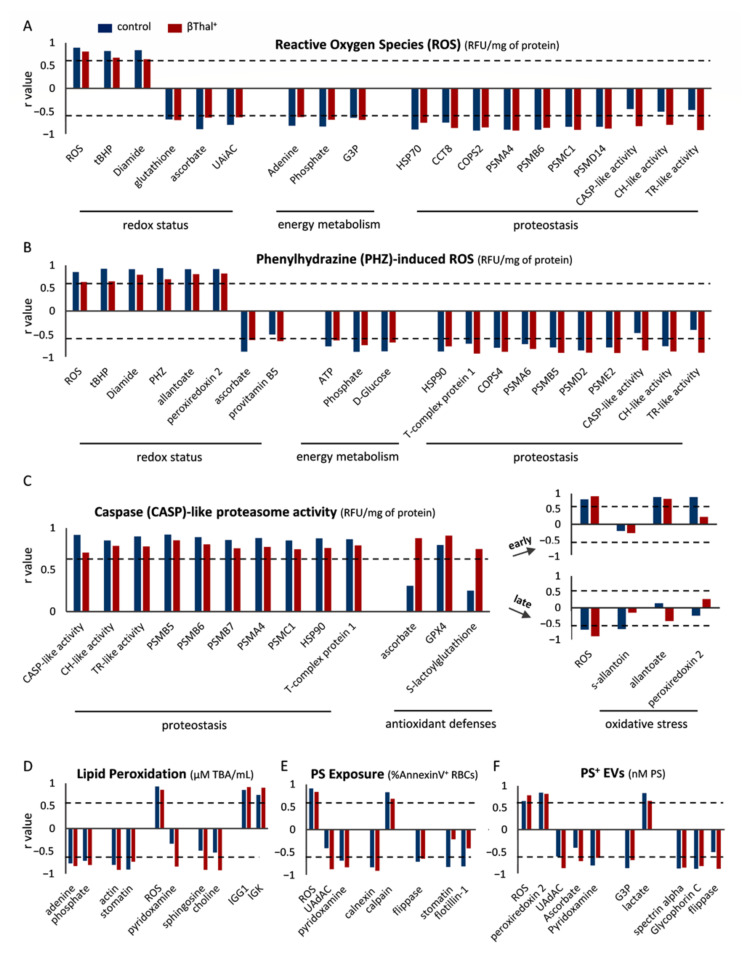
Correlations of storage variables (horizontal axis) with parameters regarding oxidative stress, proteostasis, and phosphatidylserine (PS) exposure post-mixing with plasma. Parameters correlating with (**A**) intrinsic and (**B**) phenylhydrazine-induced ROS, (**C**) caspase-like proteasome activity, (**D**) lipid peroxidation, (**E**) phosphatidylserine (PS) exposure, and (**F**) PS-positive EVs of late-stored control and beta-thalassemia trait (βThal^+^) reconstituted RBCs in thalassemic plasma (*n* = 10 per group) are shown. Similar correlations with slightly different r values emerged in all conditions tested. Dashed line: statistical threshold (*p* < 0.05). CASP: caspase, CCT: T-complex subunit, CH: chymotrypsin, COPS: COP9 signalosome subunit, G3P: glyceraldehyde 3-phosphate, GPX4: glutathione peroxidase 4, PSM: proteasome subunit, tBHP: tert-butyl hydroperoxide, TR: trypsin, UAdAC: uric-acid-dependent and UAiAC: uric-acid-independent antioxidant capacity.

Regarding ROS accumulation post-mixing, a general interconnection between intrinsic/induced ROS of stored samples and intrinsic/phenylhydrazine (PHZ)-induced ROS of reconstituted samples was observed, accompanied by a reverse correlation with antioxidant molecules, like ascorbate or glutathione (Figure 4A,B). Metabolites of energy reserve, like ATP and phosphate, and protein-control parameters, such as proteasome and T-complex subunits, also inversely correlated with intrinsic and PHZ-induced ROS. Of note, correlations with proteasomic activities exceeded the statistical threshold only in the case of βThal^+^ RBCs (Figure 4A,B). Tert-butyl hydroperoxide (tBHP)- and diamide-induced ROS post-mixing presented analogous connections (e.g., diamide-induced with diamide-induced, r = 0.837 vs. r = 0.914; T-complex protein 1 with tBHP-induced: r = −0.746 vs. r = −0.732, βThal^+^ vs. controls). Concerning caspase (CASP)-like proteasome activity, positive correlations with stored RBCs’ proteasome components and activities, chaperones, and antioxidant molecules (mainly in βThal^+^) were observed in both groups, while oxidative stress markers showed a different pattern in early versus late storage: positive correlations in the beginning (e.g., with allantoate), and negative later on (e.g., with intrinsic ROS) (Figure 4C). Similar correlations were observed in the other two activities (e.g., HSP90 with chymotrypsin (CH)-like activity, r = 0.812 vs. r = 0.875; PSMB5 with trypsin (TR)-like activity, r = 0.780 vs. r = 0.833, βThal^+^ vs. controls). Lipid peroxidation in the recipient environment was negatively associated with the levels of energy potential (e.g., adenine) and raft-related stomatin in both groups, as well as with pyridoxamine and sphingosine in the group of heterozygotes (Figure 4D). Intrinsic ROS and membrane-bound immunoglobin levels provided positive links (Figure 4D). Notably, PS exposure post-mixing, both in RBCs and EVs, was associated with RBC redox status (e.g., positive with intrinsic ROS), along with flipping-related parameters (e.g., negative with flippase) during storage. As expected, calcium homeostasis was present in the analysis of RBC PS externalization (e.g., positive with calpain), whereas energy equilibrium was reflected in the one of PS^+^ EVs (e.g., positive with lactate).

### 2.3. Transfusion to Animal Models

To examine the post-transfusion recovery of stored βThal^+^ RBCs, the newly derived RBC units were studied throughout storage to confirm that they were representative of the βThal^+^-related storage lesion profile. As expected [1], the βThal^+^ RBC units presented lower levels of storage, osmotic, mechanical, and oxidative hemolysis, either throughout storage or at mid-storage onwards, as well as increased extracellular antioxidant capacity, especially uric acid (UA)-dependent (Table S1). There was also a lower accumulation of intrinsic ROS and minor lipid peroxidation already from day 21 (Table S1).

**Figure 5:**
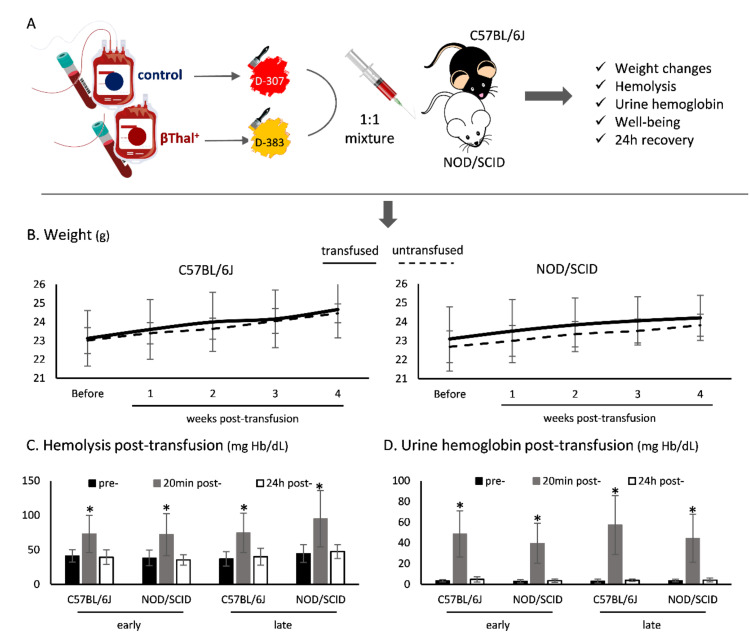
Wellbeing and hemolysis parameters of recipient mice post-transfusion with human RBCs stored under blood bank conditions. (**A**) Experimental design. (**B**) Weight changes of mice by weekly measurements post-transfusion, against non-transfused controls. Free hemoglobin in (**C**) plasma and (**D**) urine of mice before and after transfusion (*) *p* < 0.05 20 min post- versus pre- and 24 h post-transfusion. *n* = 16 per group for all measurements.

Following transfusion of a mixture of differentially labeled βThal^+^ and control RBCs to NOD.CB17-Prkdcscid/J and C57BL/6J mice (Figure 5A), there was no obvious alteration in their behavior. Specifically, animals were not lethargic but with normal mobility, showing normal food and water consumption and visible behavioral patterns concerning sociality like grooming, sniffing, and climbing. Weekly weighing showed no difference between transfused and non-transfused mice (Figure 5B), while there was an increase in free hemoglobin (Hb) in both plasma (Figure 5C) and urine (Figure 5D) 20-min post-transfusion in both immunosufficient and immunodeficient mice, but not 24 h later.

To be sure that the observed hemolysis did not differ between the two donor groups (and hence did not influence the results of post-transfusion recovery), we infused animals of the two genetic backgrounds with RBCs from controls and heterozygotes separately. No difference was observed neither in plasma (e.g., late-stored, 20 min in C57BL/6J: 81.37 ± 38.55 vs. 85.62 ± 31.57; in NOD.CB17-Prkdcscid/J: 92.55 ± 32.12 vs. 87.32 ± 28.48, βThal^+^ vs. controls) nor in urine free Hb (e.g., late-stored, 20 min in C57BL/6J: 48.52 ± 19.35 vs. 42.86 ± 21.7; in NOD.CB17-Prkdcscid/J: 40.32 ± 26.70 vs. 51.28 ± 32.52, βThal^+^ vs. controls).

**Figure 6:**
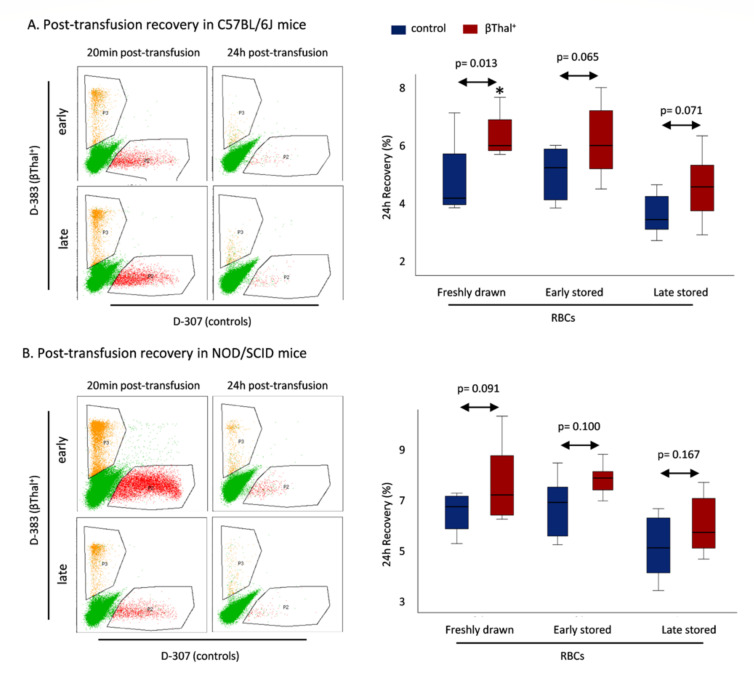
Recovery of human RBCs 24 h post transfusion in recipient-mice. Indicative flow cytometry dot plot images and the levels of post-transfusion recovery are shown in (**A**) C57BL/6J and (**B**) NOD.CB17-Prkdcscid/J mice. (*) *p* < 0.05, beta-thalassemia trait (βThal^+^) vs. control transfused RBCs (*n* = 8 per group).

Moving on to post-transfusion recovery, there was a universal trend for higher levels in the RBCs of βThal^+^ in both C57BL/6J (Figure 6A) and NOD.CB17-Prkdcscid/J (Figure 6B) mouse recipients.

While it has been previously shown that the two dyes used do not label differently cells of distinct donor groups and do not alter the transfusion outcome (as evidenced by transfusion of unstained RBCs to mice expressing the green fluorescent protein—GFP [22]), since βThal^+^ RBCs have not been tested in post-transfusion recovery experiments yet, we wanted to check for this feature in our samples. Indeed, comparable trends for higher recovery in heterozygotes were observed when βThal^+^ and control RBCs were reversely labeled (Table S2). It should be noted that if the two labeling experiments (which provide equivalent results) are taken together (*n* = 16 transfusion events), the trends and sporadic significant differences, observed in Figure 6 and Table S2, universally surpass the threshold of statistical significance (e.g., early-stored RBCs in NOD.CB17-Prkdcscid/J: 7.57 ± 1.07 vs. 6.54 ± 1.16, *p* = 0.016; late-stored RBCs in C57BL/6J: 4.83 ± 1.19 vs. 3.89 ± 0.75, *p* = 0.012, βThal^+^ vs. controls).

### 2.4. Correlations of Recovery with Storage Physiology

**Figure 7:**
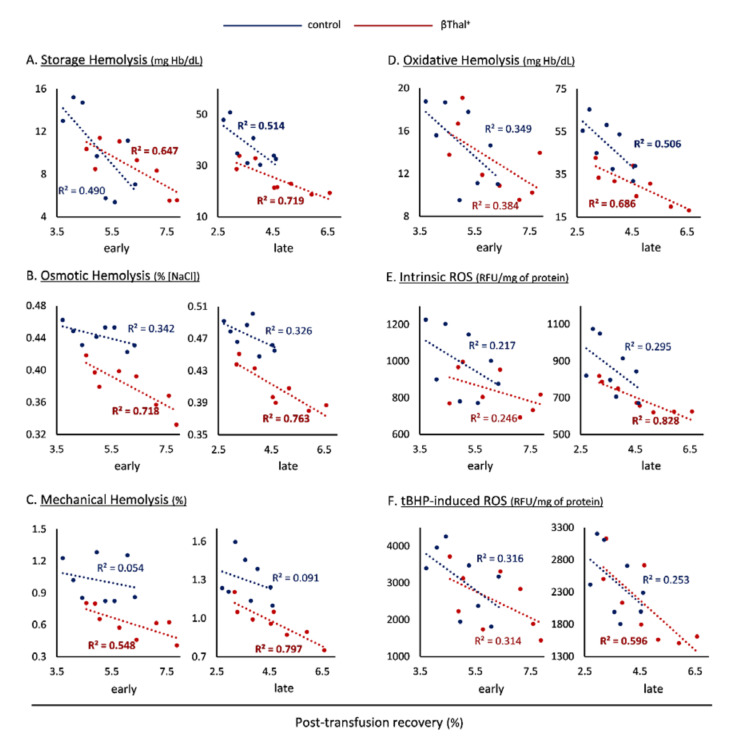
Correlations between physiological parameters of stored RBCs and post-transfusion recovery in C57BL/6J mice. Correlations of recovery with (**A**) storage, (**B**) osmotic, (**C**) mechanical, and (**D**) oxidative hemolysis, as well as with (**E**) intrinsic and (**F**) tBHP-induced ROS are shown for beta-thalassemia trait (βThal^+^) and control RBCs (*n* = 8 per group). R^2^ > 0.5 for *p* < 0.05. Similar results, with slightly different R^2^ values emerged in NOD.CB17-Prkdcscid/J recipient mice.

Our next and final step was to search for possible correlations between the storage physiology and the post-transfusion recovery of βThal^+^ and control RBCs. Interestingly, storage (Figure 7A), osmotic (Figure 7B), and mechanical (Figure 7C) hemolysis demonstrated significant negative correlations with 24 h recovery of βThal^+^ RBCs, in both early and late storage, whereas only spontaneous hemolysis of late storage exceeded the significance threshold in the control group (Figure 7A). Among the oxidative-stress-related variables, while end-of-storage oxidative hemolysis was inversely associated with the recovery of both groups’ transfused RBCs (Figure 7D), intrinsic (Figure 7E) and tBHP-induced (Figure 7F) ROS exhibited the same link only in the case of heterozygotes.

## 3. Discussion

Previous works from our team have demonstrated that stored RBCs from blood donors with beta-thalassemia traits are superior to the average control regarding (a) hemolysis (spontaneous or stress-induced); (b) removal signaling; (c) redox equilibrium [1]; (d) preservation of membrane and cytoskeleton components [6]; and (e) membrane proteo-vigilance served by a complex network of active proteasomes and recruited “protect, repair, destroy or sacrifice” proteins [8,23]. We hereby validate and expand on these findings from βThal^+^ stored RBCs, by reporting for the first time maintenance of their outstanding resistance to lysis in a recipient-mimicking environment in vitro, as well as the potential for higher post-transfusion recovery in xenobiotic animal models of transfusion. Furthermore, our results give hints about aspects of RBC physiology, such as hemolysis, energy/redox equilibrium, membrane/cytoskeleton properties, and proteostatic elements, that link the storage and post-storage/post-transfusion behaviors of RBCs.

### 3.1. Stored RBC Features in Recipient Plasma and Temperature

The current results support that βThal^+^ RBCs are characterized by favorable hemolysis from the time of donation (mainly fragility indices), throughout storage [1] and even within a potential recipient’s environment (Figure S1). The fact that these profiles are evident in both plasma conditions indicates that βThal^+^ RBCs’ resistance to lysis is an intrinsic characteristic, not affected by common soluble plasma factors and ambient temperature. A plausible explanation for this resistance to rupture [24] is the reduced cellular volume and increased surface:volume ratio of βThal^+^ RBCs [25]. Moreover, it might be connected to their unique cytoskeleton [6], as evidenced by the massive correlation outcomes between fragility indices and several cytoskeletal components. A recent genome-wide association study of the REDS-III program reinforces our findings, especially regarding osmotic fragility: genes known to modulate RBC membrane organization, like band-3, myosin and spectrin, have been found associated with the osmotic hemolysis of stored RBCs [7]. The same was also true for donors carrying common single nucleotide polymorphisms in the *N*-terminus of band-3 [26]. The specialized spectrin-based RBC cytoskeleton and its numerous linkages to membrane proteins allow the cell to reversibly deform while passing through the narrow capillaries, while a variety of mutations in the genes of the proteins involved lead to hemolytic anemias in humans [27]. Non-muscle myosin II has been also associated with the control of the curvature and deformability—a biophysical marker of RBC fragility [28]—of cells with either actin- or spectrin/actin-based cytoskeletons [29,30]. Altogether; (a) the higher levels and/or stability of several structural proteins in βThal^+^ RBCs throughout storage [6], (b) the lower levels of post-mixing hemolysis, fragility indices and microvesiculation (indirectly measured) in the same cells, and (c) the mutual—amongst the two groups—negative correlation between (a) and (b), tempt us to assume that the construction of βThal^+^ RBCs is one of the main reasons behind their resistance to lysis. In addition, piezo-1, a mechanosensor that regulates RBC shape through ion-related volume reduction, was found to be decreased in the same donors [6], in close correlation with mechanical (in both controls and heterozygotes) and in association with osmotic hemolysis [7]. It is very enticing to hypothesize that the microcytic βThal^+^ RBCs are less susceptible to shear stress in the circulation, and thus minimal signal transduction through piezo-1 molecules is required. Whether decreased levels of piezo-1 molecules are genetically determined in these cells remains unclear, however, association/correlation with osmotic and mechanical fragilities might be the reason behind the observed preferential crosstalk between the two fragility indices only in stored/reconstituted heterozygous erythrocytes.

In the same context, reconstituted βThal^+^ RBCs possess enhanced proteasome activity, which nonetheless presents a universal correlation with the increased storage levels of proteasome (components and activities) and chaperones compared to controls [6,8]. Moreover, antioxidant defense molecules were found to be tightly connected to βThal^+^ proteasome activity post-mixing, highlighting once again the closer connection between redox balance and proteostasis in these subjects [8]. It appears that the storage levels of “protect, repair or destroy” components are not neutral to post-storage proteasome activities; a finding that may be relevant to transfusion outcomes. The interesting change in the correlation sign observed between the storage levels of oxidative stress and post-mixing proteasome activity in early versus late storage further supports—and expands to a post-storage environment—the hypothesis that proteasome machinery is both effective against [31] and affected by [32] the oxidative burden [8]. This interplay between redox equilibrium and protein quality control was anticipated. It has been shown before by protein–protein interactomes and bioinformatics analyses [33,34], and it is currently observed in both directions: proteostasis is not only affected by oxidative stress, but it seems to protect the reconstituted cells from oxidative lysis and ROS accumulation. Interestingly, lower oxidative RBC lysis, after treatment with the Hb-targeting phenylhydrazine, was observed in the proteasome enriched βThal^+^ samples, implying a role for the proteasome in the degradation of oxidized Hb [35].

Naturally, the redox and energy equilibrium of stored RBCs affected all tested post-mixing parameters in both groups. It is widely established that oxidative stress lies behind storage lesion phenotypes [36], while antioxidant molecules of stored RBCs have been found to correlate with the 24 h post-transfusion recovery in mice [37]. In accordance, our in vitro experiments revealed interesting correlations of redox factors with RBC physiology. The oxidative burden of the cells, mainly shown through ROS accumulation, leads to fragile cells that are more prone to oxidative damage and surface exposure of removal signals in both groups post reconstitution, while ROS and oxidative hemolysis levels post-storage are proportional to those before reconstitution. The first observations were expected, since the oxidative stress generated under storage conditions insults key structural and functional RBC components leading to PS exposure [36] and reduction of deformability [28,38]. However, to our knowledge, this is the first time that measures of oxidative stress in stored RBCs have been shown to be conserved in a post-storage state. On the contrary, several antioxidant molecules of stored RBCs counteract to protect them post-storage. For instance, glutathione peroxidase 4 (GPX4) levels anticorrelate with hemolysis, and extracellular antioxidant capacity with ROS accumulation and concentration of procoagulant EVs. Both findings have been previously observed during storage [39,40], so it seems that these antioxidants continue to protect stored RBCs after “re-addition” of plasma. Finally, pyridoxamine, previously shown to (a) ameliorate oxidative and ion transport defects in RBCs [41], and (b) be more abundant in βThal^+^ stored erythrocytes [1], showed many negative correlations with an array of parameters, a great part of which were favorable in the group of heterozygotes post-mixing (e.g., hemolysis and lipid peroxidation). Stored RBCs are characterized by rapid depletion of energy, even under hypothermic conditions. Lactate, a biomarker of stored RBC age [42], accumulates during storage leading to pH alteration and metabolism inhibition, hence its positive correlation with a variety of lesions. When found in an environment closer to “normal”, like the one of our model, RBC enzymes can restart working at their natural pace, as evidenced by the rapid replenishment of 2,3-diphosphoglycerate [43] and ATP [44] post-transfusion. Nonetheless, the correlations between storage energy metabolism/equilibrium and almost every parameter tested post-reconstitution indicate that better preservation of energy reservoirs benefits even more the cell physiology post-mixing. To support this, stored erythrocytes with greater ATP levels present a higher viability post-transfusion [45,46]. Energy metabolites have been also shown to be beneficial for RBC integrity during storage [47]. The other side of the coin, AMP, a low energy signal also present in our correlation outcomes, is a precursor of hypoxanthine, a metabolite shown to anticorrelate with post-transfusion recovery in both mice and men [48].

### 3.2. Transfusion to Animal Models

The discrete physiology of βThal^+^ RBCs that has been proven to be beneficial during storage and post reconstitution, might be the underlying force for the observed trend—or statistical significance if all transfusion replicates/events are taken into account—for superior post-transfusion recovery in vivo. This strong trend, which was not attributed to acute clearance due to hemolytic reactions (evidenced by similar between-groups levels of plasma and urine Hb), arose in both immunosufficient and immunodeficient mice, with the levels of 24 h recovery being higher in the latter case, as previously reported [49]. Correlations that predominated in βThal^+^ subjects, mainly between hemolysis (with or without additional stresses) and post-transfusion recovery, further support our claim. Interestingly, a recent study investigating obese blood donors also revealed an inverse association of storage and osmotic hemolysis with post-transfusion recovery [17]. The preferential linkage of fragility indices with RBC survival in the group of heterozygotes is extremely fascinating. It is known that poorly deformable RBCs are retained in the spleen [50], while replication of the inter-endothelial slits in vitro via microfluid devices suggested that the mechanically aged RBCs are susceptible to phagocytosis, showing in parallel loss of proteins involved in cytoskeleton architecture, cell metabolism, antioxidant protection, and microvesiculation of the membrane [51]. Of note, rapid clearance of non-reversible micro-erythrocytes decreases transfusion recovery [52]; a finding consistent with the higher levels of circulating βThal^+^ RBCs in the blood stream of our transfused mice, since these cells are reported to resist non-reversible transformation during middle/late storage [6]. In this context, it should not be omitted that the size of transfused RBCs (lower in the case of βThal^+^) has been found to be reversibly associated with recovery in xenobiotic models of transfusion. Nevertheless, (a) our study design involved selection of eligible βThal^+^ blood donors without extremely low MCV values (73.3 ± 6.8 vs. 82.6 ± 2.1 fL, βThal^+^ vs. controls) in order to ameliorate the size effect upon RBC clearance; (b) the tendency remained in favor of heterozygotes even in transfused pairs of βThal^+^-control RBCs with minimal MCV variation between each other; and (c) it’s worth remembering that RBCs experience excessive mechanical stress during their passage through the human spleen slits of very small diameter (0.5–1.0 μm [53]). The forces deforming RBCs in a mouse environment are more intense. However, our results have the potential to be expanded in a human setting, since βThal^+^ erythrocytes will still experience less mechanical pressure compared to control: they have both lower size and lower levels of sublethal lesions, including susceptibility to lysis and oxidative insults. What can be argued is that survival in the mouse circulation may not simulate the one in transfused humans, and that absolute values of xenobiotic 24 h RBC recovery cannot be normalized to the gold-standard of transfusion in humans, namely >75%. Nevertheless, these animal models have provided valuable evidence, though not definite conclusions, in studies of other donor backgrounds, such as sickle cell trait [22] and the REDS-III obese cohort [17]. Even though concrete evidence of RBC recovery “success” can be only provided in vivo (in animal models and mainly, in human) our findings further highlight the usefulness of the currently used in vitro models in the field: several correlations between storage and post-storage states were found in common. To support this, G6PD deficient donors were at first characterized as “bad donors” by using a very alike in vitro model [13], and subsequently validated as such in a clinical trial setting [16].

## 4. Materials and Methods

### 4.1. Biological Samples and Blood Unit Preparation

Twenty leukoreduced units of packed RBCs in citrate-phosphate-dextrose (CPD)/saline-adenine-glucose-mannitol (SAGM) (10 βThal^+^ and 10 controls), the storage physiology, metabolism and proteome of which have been analyzed in previous studies of this project [1,6,8], were selected to evaluate the physiological parameters of stored RBCs in recipient plasma at body temperature in vitro. Plasma was isolated from freshly drawn blood donated by 10 healthy volunteers and 10 transfusion-dependent beta-thalassemia major patients (shortly before transfusion), in citrate vacutainers. Sixteen (eight per group) additional RBC units (leukoreduced; CPD/SAGM) were used for the evaluation of RBC recovery in vivo by using animal models of transfusion, following validation of the storage profile of βThal^+^ RBCs. The study was approved by the Ethics Committees of the Department of Biology, School of Science, NKUA and of the Biomedical Research Foundation of the Academy of Athens (BRFAA). Investigations were carried out upon donor consent, in accordance with the principles of the Declaration of Helsinki.

### 4.2. Exposure of Stored RBCs to Human Plasma at Body Temperature

For the simulation of the effects of body temperature and recipient plasma components upon stored RBCs, an in vitro model was used, as extensively described in the past [13]. Briefly, early (storage day <4) and late (storage day >39) stored RBCs from heterozygotes and controls were incubated for 24 h at 37 °C and in 5% CO_2_-air, following reconstitution in freshly drawn plasma from potential recipients (controls and beta-thalassemia major patients) mixed with the same unit’s supernatant, in a ratio respective to transfusion of two blood units. To avoid settling, the samples were under constant gentle agitation throughout the incubation period. Measurements of hemolysis, ROS concentration, phosphatidylserine exposure, membrane lipid peroxidation, and proteasome activity were performed in the reconstituted samples.

### 4.3. Animal Model of Transfusion

All experiments were performed in the Biomedical Research Foundation of the Academy of Athens (BRFAA), and the study protocol was approved by the Department of Agriculture and Veterinary Service of the Prefecture of Athens (Permit Number: 534915, 23 July 2020). A total of 32 immunodeficient NOD.CB17-Prkdcscid/J and wild type C57BL/6J male mice, 8–12 weeks old (16 per genetic group), were used as a xenobiotic model of transfusion to evaluate the 24 h RBC recovery, as previously described [22,49,54] and extensively analyzed in the Supplementary File. Freshly drawn, early- (<4 days) and late-stored (>39 days) RBCs were labeled with the lipophilic dyes D-383 (1,1’-Didodecyl-3,3,3’,3’-Tetramethylindocarbocyanine Perchlorate; for βThal^+^) and D-307 (1,1’-Dioctadecyl-3,3,3’,3’-Tetramethylindodicarbocyanine Perchlorate; for controls), as per manufacturer’s instructions (Invitrogen, Carlsbad, CA, USA), prior to their infusion as a 1:1 mixture (~55% hematocrit with sterile PBS 310mOsm) into 16 recipient-mice (8 per genetic group) by intravenous injection in the tail vein. The same procedure was performed in another set of 16 mice, with the opposite RBC labeling. Blood sampling via the facial vein was performed one day before, 20 min (100% recovery) and 24 h (24 h RBC recovery) post-infusion to evaluate (a) the post-transfusion recovery through flow cytometry (FACSAria IIu/Diva software, BD Pharmingen, San Jose, CA, USA), and (b) the levels of intravascular hemolysis through spectrophotometry. The initial measurement was made 20 min post-transfusion in order to effectively handle the large number of animals and provide reliability in the comparative measurements. Urine was also collected at the same time points to evaluate the hemoglobin (Hb) levels.

### 4.4. Hemolysis Parameters

Spontaneous hemolysis was evaluated in the supernatant of stored units and reconstituted samples through spectrophotometry [55], followed by the Allen correction. Osmotic hemolysis was assayed by exposing the samples in ascending concentrations of NaCl and calculating the mean corpuscular fragility (MCF) index (i.e., % NaCl at 50% hemolysis). Rocking with stainless steel beads for 1 h was used to implement a mechanical stimulus. The released Hb was measured in the supernatant of rocked and non-rocked samples to infer only the mechanically induced hemolysis. Lastly, oxidative hemolysis levels were evaluated after treatment of RBCs with phenylhydrazine (PHZ; 17 mM) for 1 h at 37 °C [1].

### 4.5. Oxidative Stress-Related Parameters

ROS accumulation was detected with and without additional oxidative stimuli (tert-butyl hydroperoxide -tBHP, diamide and PHZ) through fluorometry (VersaFluor, BIO-RAD, Hercules, CA, USA), by using the redox-sensitive and membrane permeable 5-(and-6)-chloromethyl-2′,7′-dichloro-dihydro-fluorescein diacetate, acetyl ester (CM-H_2_DCFDA, Invitrogen, Molecular Probes, Eugene, OR, USA), as thoroughly reported before [1]. The ferric reducing antioxidant power (FRAP) assay [56] was performed in the supernatant of the units, in the absence or presence of uricase treatment, to determine the total (TAC), uric-acid-dependent (UAdAC) and uric-acid-independent (UAiAC) antioxidant capacity. For membrane lipid peroxidation assessment, RBCs were mixed with 20% trichloroacetic acid, and the supernatant (lipid part) of these samples was then treated with 0.67% thiobarbituric acid (TBA, 50 min at 90 °C). TBA and malondialdehyde (MDA; biomarker of lipid peroxidation) form a chromogenic complex that was measured at 532 nm [1]. All oxidative reagents, uricase, TCA and TBA were purchased from Sigma Aldrich (Munich, Germany).

### 4.6. Proteasome Activity

Caspase (CASP)-like, trypsin (TR)-like and chymotrypsin (CH)-like proteasome activities were determined in stored red blood cells through fluorometry [57]. Briefly, 120–200 μg of packed RBCs in 20 mmol/L Tris-HCl (pH 7.5–8.0) were incubated for 1.5 h to 3 h at 37 °C with the substrates: Suc-Leu-Leu-Val-Tyr-aminomethylcoumarin (AMC) for CH-like, z-Leu-Leu-Glu-AMC for CASP-like and Boc-Leu-Arg-Arg-AMC for TR-like activity. The experiment was also performed in the presence of proteasome inhibitors (10–20 µM bortezomib for the CH- and CASP-like activities, 200 µM MG-132 for the CH-like activity, and 100 µM lactacystin for the TR-like activity) to subtract the unspecific proportion (4–8%) of the total fluorescence levels. All substrates and inhibitors were procured from Enzo Life Sciences (New York, NY, USA).

### 4.7. Phosphatidylserine Exposure on RBCs and Extracellular Vesicles (EVs)

Phosphatidylserine (PS) exposure was estimated by multicolor flow cytometry following labeling of RBCs with phycoerythrin (PE)-Annexin V and fluorescein isothiocyanate (FITC)-conjugated anti-CD235 antibody (BD Pharmingen, San Jose, CA, USA), while an ELISA kit (Zymuphen MP-activity, Hyphen BioMed, Neuvillesur-Oise, France) was used to measure the PS-positive (procoagulant) EVs, per the manufacturer’s specifications and as previously described [58].

### 4.8. Statistical Analysis

All physiological experiments were performed in triplicate. For statistical analysis, SPSS (Version 22.0, IBM Hellas, Athens, Greece, administered by NKUA) computer software was used. After testing for normal distribution profile and presence of outliers (Shapiro–Wilk test and detrended normal Q-Q plots), intergroup differences were evaluated by repeated measures analysis of variance (ANOVA). Differences in post-transfusion recovery levels were analyzed by using independent *t*-test. Pearson’s and Spearman’s tests were performed to assess correlations between parameters. Regarding correlation analysis of the in vitro model data, we considered only the repeatable significant correlations between stored and reconstituted RBCs reliable, i.e., the ones evident at both time points and “recipient plasma” backgrounds. Receiver operating characteristic (ROC) curves were used to identify parameters strongly indicative of βThal^+^ status in reconstituted RBCs. The proteomic, metabolomic, and physiological data of blood units used for the correlation analysis of Figure 3 and Figure 4 originate from previous works of the βThal^+^ donors project [1,6,8]. *p* < 0.05 was considered statistically significant.

## 5. Conclusions

Heterozygotes for mutations in the beta-globin gene are eligible donors whose RBCs are less susceptible to storage lesions and less fragile after re-addition of plasma at body temperature. Their discrete physiology, characterized by a notable cytoskeleton, membrane proteo-vigilance and resistance to lysis, seems to underlie these observed superiorities and probably the currently reported better post-storage performance, both in vitro and in vivo. It appears as if βThal^+^ RBCs “check” both gold standards of transfusion, namely end-of-storage hemolysis and 24 h post-transfusion recovery (at least in a xenobiotic mouse model). No doubt, these results need to be expanded and validated in a human setting through clinical trials, to also assess the Hb increment. Although it is feared that carriers of Hb mutations might show a drawback in this parameter, we strongly believe that this would not be the case. Many βThal^+^ subjects eligible for blood donation (including those reported in the current study) have slightly (rather than significantly) lower than average intracellular Hb levels, and at the same time their stored RBCs show significantly lower hemolysis, surface removal signaling, and sublethal lesions. Consequently, Hb increment post transfusion with βThal^+^ RBC units is expected to be at least equal, if not superior, to that observed following transfusion with control units. We are looking forward to future studies that will focus on this hypothesis.

## Data Availability

All physiological data presented in this study are available upon request.

## Supplementary Materials

The following are available online at www.mdpi.com/article/10.3390/ijms222212281/s1.
