# Supplementary material for: The Post-Storage Performance of RBCs from Beta-Thalassemia Trait Donors Is Related to Their Storability Profile
Source: Int J Mol Sci. 2021 Nov 13;22(22):12281. doi: 10.3390/ijms222212281 (PMC8619127; doi:10.3390/ijms222212281)
Supplement: Supplementary file 1 [file ijms-22-12281-s001.zip › Supplementary_Methods.pdf]

Supplementary Methods

Animal Model of Transfusion

All experiments were performed in the Biomedical Research Foundation of the Academy of Athens (BRFAA). The facility is registered as "breeding" and "experimental" facility (Reg. Numbers: EL BIO 01 and EL 25 BIO 03, respectively) according to the Greek Presidential Decree 56/2013, which harmonizes National Legislation with the European Directive 2010/63 on the protection of animals used for scientific purposes. All applicable National guidelines for the care and use of animals were followed. The study protocol was approved by the Department of Agriculture and Veterinary Service of the Prefecture of Athens (Permit Number: 534915/23-07-2020). Cages were kept in the same animal room with HEPA filtered air supply, 15 ACH, light intensity of 300 lux measured one meter above the floor in the middle of the room and color temperature of 4100 K as well as positive air pressure of 0.6 Pa within the room. Room conditions were continuously monitored through the central Building Management System (BMS) of the animal facility.

Animals were bred and maintained in a specific pathogen-free, temperature- and humidity-regulated unit (21 ± 2 °C; 55% ± 10%), and a 12/12 h light/dark cycle with lights off at 19:00 h and no twilight period. Mice were housed in individually ventilated cages (IVC) (Seal Safe 1284 L, H-Temp™, Techniplast, Varese, Italy) receiving 70 air changes per hour, at a stocking density of 4–5 mice per cage unless otherwise stated [caging dimensions (L × W × H): 365 × 207 × 140 mm, floor area = 530 cm^2]. All mice had ad libitum access to filtered tap water in drinking bottles and a vacuum-packed pelleted rodent chow that contained 18.5 % protein, 5.5 % fat, 4.5 % fiber, and 6 % ash (4RF22, Mucedola, Milan, Italy). The bedding in each cage comprised of ~250 g of autoclaved corncob bedding (Rehofix MK 2000, J. Rettenmaier & So, Rosenberg, Germany). The cages were cleaned and autoclaved once a week. All animals were clinically evaluated by an experienced veterinarian daily. Food and water consumption along with weight, mobility and social behavior was recorded and evaluated every week till the end of the experimentation.

A total of 32 immunodeficient NOD.CB17-Prkdcscid/J and wild type C57BL/6J male mice, 8-12 weeks old, (16 per genetic group) were used as a xenobiotic model of transfusion to evaluate the 24h RBC recovery, as previously described [1-3]. We used only male animals to reduce the variability of the outcome caused by sex-differences and to, thereby, decrease group sizes. Freshly drawn, early- (< 4 days) and late-stored (>39 days) RBCs were labeled with the lipophilic dyes D-383 (1,1'-Didodecyl-3,3,3',3'-Tetramethylindocarbocyanine Perchlorate; for $\beta Thal^+$) and D-307 (1,1'-Dioctadecyl-3,3,3',3'-Tetramethylindodicarbocyanine Perchlorate; for controls) as per manufacturer's instructions (Invitrogen, Carlsbad, CA, USA), prior their infusion as a 1:1 mixture (~55% hematocrit with sterile PBS 310mOsm) into 16 recipient-mice (8 per genetic group) by intravenous injection in the tail vein. The transfused volume (~200µL) is respective to two blood units (one of each donor). The same procedure was performed in another set of 16 mice, with the opposite RBC labeling (i.e., D-383 for control and D-307 for $\beta Thal^+$) in order to ensure that there was not differential dying between the two donor groups. Blood sampling via the facial vein was performed one day before, 20 minutes (100% recovery) and 24h (24h RBC recovery) post-infusion to evaluate (a) the post-transfusion recovery through flow cytometry

(FACSAriaIIu/Diva software, BD Pharmingen, San Jose, CA) and (b) the levels of intravascular hemolysis through spectrophotometry. Urine was also collected at the same time points to evaluate the hemoglobin (Hb) levels. Mice are compelled to urinate through application of abdominal pressure by grasping them from behind and seizing the skin around the neck. An additional group of 20 mice (10 of each genetic group) were used for further validation of the Hb concentration in plasma and urine post-transfusion with RBCs from each donor group without sample mixing.

References

1. Osei-Hwedieh, D.O.; Kanias T.; Croix C.S.; Jessup M.; Xiong Z.; Sinchar D.; Franks J.; Xu Q.; E M.N.; Sertorio J.T.; Potoka K.; Binder R.J.; Basu S.; Belanger A.M.; Kim-Shapiro D.B.; Triulzi D.; Lee J.S.; Gladwin M.T. Sickle Cell Trait Increases Red Blood Cell Storage Hemolysis and Post-Transfusion Clearance in Mice. *EBioMedicine* **2016**, *11*, 239-48. 10.1016/j.ebiom.2016.08.006
2. Blessinger, S.A.; Tran J.Q.; Jackman R.P.; Gilfanova R.; Rittenhouse J.; Gutierrez A.G.; Heitman J.W.; Hazegh K.; Kanias T.; Muench M.O. Immunodeficient mice are better for modeling the transfusion of human blood components than wild-type mice. *PLoS One* **2020**, *15*, e0237106. 10.1371/journal.pone.0237106
3. Hod, E.A.; Zhang N.; Sokol S.A.; Wojczyk B.S.; Francis R.O.; Ansaldi D.; Francis K.P.; Della-Latta P.; Whittier S.; Sheth S.; Hendrickson J.E.; Zimring J.C.; Brittenham G.M.; Spitalnik S.L. Transfusion of red blood cells after prolonged storage produces harmful effects that are mediated by iron and inflammation. *Blood* **2010**, *115*, 4284-92. 10.1182/blood-2009-10-245001
