# Supplementary material for: The Post-Storage Performance of RBCs from Beta-Thalassemia Trait Donors Is Related to Their Storability Profile
Source: Int J Mol Sci. 2021 Nov 13;22(22):12281. doi: 10.3390/ijms222212281 (PMC8619127; doi:10.3390/ijms222212281)
Supplement: Supplementary file 1 [file ijms-22-12281-s001.zip › Table S1.pdf]

Table S1. Storage differences in physiological parameters between control and βThal⁺ red blood cells (RBCs)

Parameters	Day 7		Day 21		Day 42	
	Control	βThal$^+$	Control	βThal$^+$	Control	βThal$^+$
Storage Hemolysis (mg Hb/dL)	10.47±3.78	8.70±2.24	22.8±4.70	17.10±3.67*	37.22±7.69	25.66±5.61*
Osmotic Hemolysis (% NaCl)	0.443±0.011	0.381±0.029*	0.474±0.010	0.408±0.036*	0.480±0.019	0.409±0.032*
Mechanical Hemolysis (%)	1.025±0.190	0.623±0.147*	1.148±0.195	0.756±0.082*	1.302±0.181	0.977±0.146*
Oxidative Hemolysis (mg Hb/dL)	14.57±3.85	13.26±3.39	29.88±7.42	19.34±6.50*	48.60±12.53	29.93±8.81*
Extracellular TAC (μM Fe^{2+})	418±60	523±95*	392±36	472±103	355±52	380±63
Extracellular UAdAC (μM Fe^{2+})	239±29	358±69*	231±38	296±51*	161±45	155±64
Extracellular UAiAC (μM Fe^{2+})	178±59	165±48	161±34	176±96	194±39	235±35
Intrinsic ROS (RFU/mg of protein)	980±201	840±133	1084±102	942±110*	870±145	694±72*
tBHP-induced ROS (RFU/mg of protein)	3043±964	2520±832	3994±1217	2987±1233	2435±571	2112±610
Diamide-induced ROS (RFU/mg of protein)	2067±612	1888±679	2198±621	1983±633	1933±688	1463±591
PHZ-induced ROS (RFU/mg of protein)	15751±5380	18152±5552	16492±4238	16713±3425	14211±5344	15407±6125
Lipid Peroxidation (μM TBA/ mL RBC)	35.16±5.26	30.57±3.19	43.32±6.04	36.10±4.58*	48.41±5.12	41.30±5.16*

Data are presented as mean±SD. (*) $p<0.05$. TAC: total antioxidant capacity; UAdAC: uric acid-dependent antioxidant capacity; UAiAC: uric acid-independent antioxidant capacity; ROS: reactive oxygen species; RFU: relative fluorescence units; tBHP: tert-butyl hydroperoxide; PHZ: phenylhydrazine; TBA: thiobarbituric acid
