# Supplementary material for: The Post-Storage Performance of RBCs from Beta-Thalassemia Trait Donors Is Related to Their Storability Profile
Source: Int J Mol Sci. 2021 Nov 13;22(22):12281. doi: 10.3390/ijms222212281 (PMC8619127; doi:10.3390/ijms222212281)
Supplement: Supplementary file 1 [file ijms-22-12281-s001.zip › Table S2.pdf]

Table S2. Post-transfusion recovery differences between control and βThal$^+$ red blood cells (RBCs) with reverse dying.

	Freshly drawn RBCs		Early storage		Late storage	
	Control	βThal$^+$	Control	βThal$^+$	Control	βThal$^+$
Recovery in C57BL/6J mice (%)	4.36±1.32	5.59±1.06	4.83±0.89	5.96±1.19*	4.10±0.82	5.05±1.22
	p=0.059		p=0.049		p=0.091	
Recovery in NOD/SCID mice (%)	6.26±0.97	7.03±1.35	6.27±1.08	7.29±0.91	5.43±1.30	6.53±1.33
	p=0.224		p=0.085		p=0.116	

Data are presented as mean±SD. (*) p<0.05.
