# Supplementary material for: The Post-Storage Performance of RBCs from Beta-Thalassemia Trait Donors Is Related to Their Storability Profile
Source: Int J Mol Sci. 2021 Nov 13;22(22):12281. doi: 10.3390/ijms222212281 (PMC8619127; doi:10.3390/ijms222212281)
Supplement: Supplementary file 1 [file ijms-22-12281-s001.zip › Figure S1.pdf]

Figure S1

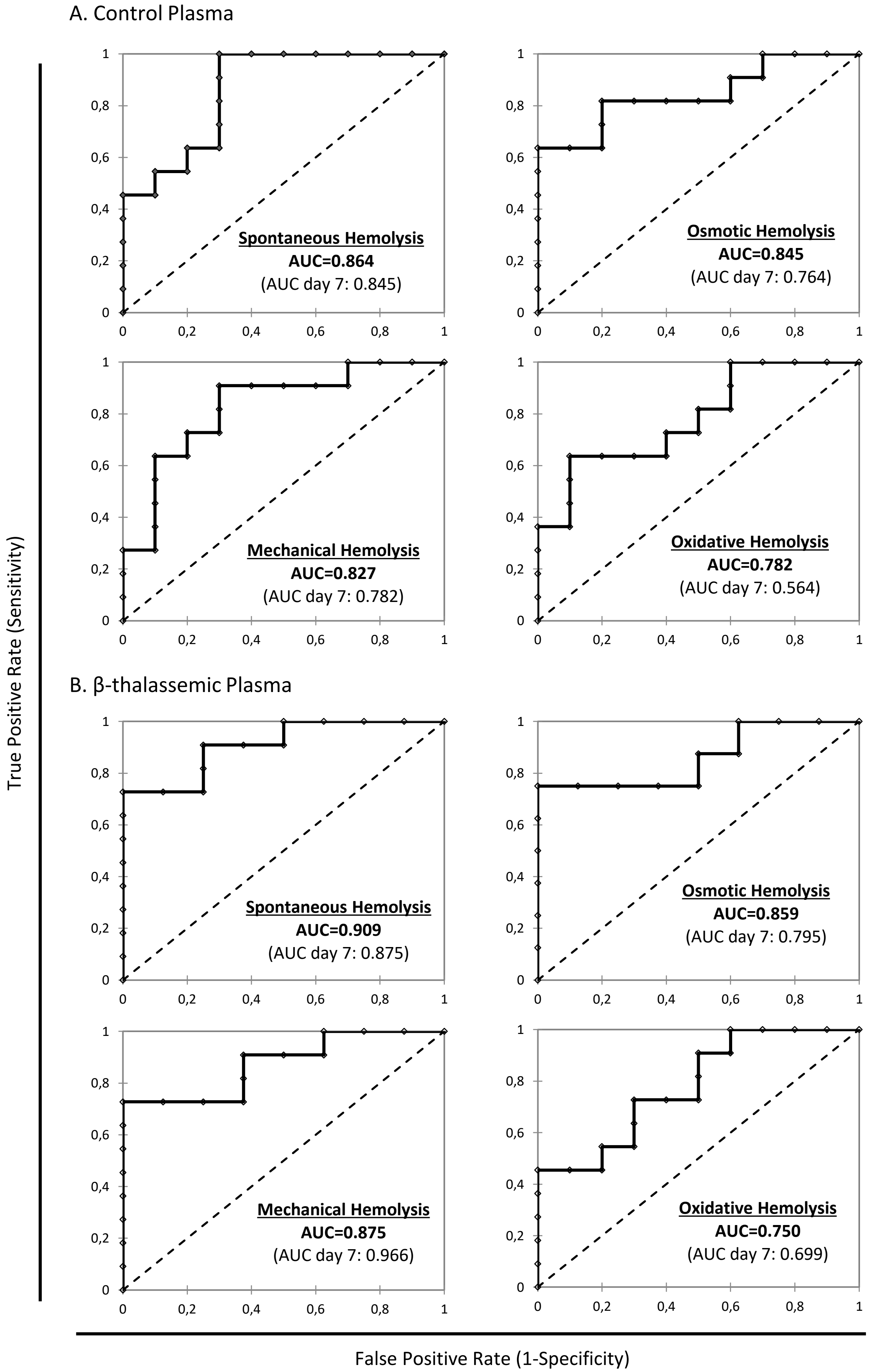

Figure S1. Receiver operating characteristic (ROC) curves that distinguish beta-thalassaemia minor (βThal^{+}) reconstituted RBCs. ROC curves for hemolysis parameters post reconstitution of stored RBCs in (A) control and (B) β-thalassemic major plasma. Only curves for late storage are shown; similar statistically significant results were observed for early storage, except for oxidative hemolysis. AUC: area under the curve.
